# Integrative analysis of transcriptomics and metabolomics to reveal the melanogenesis pathway of muscle and related meat characters in Wuliangshan black-boned chickens

**DOI:** 10.1186/s12864-022-08388-w

**Published:** 2022-03-02

**Authors:** Tengfei Dou, Shixiong Yan, Lixian Liu, Kun Wang, Zonghui Jian, Zhiqiang Xu, Jingying Zhao, Qiuting Wang, Shuai Sun, Mir Zulqarnain Talpur, Xiaohua Duan, Dahai Gu, Yang He, Yanli Du, Alsoufi Mohammed Abdulwahid, Qihua Li, Hua Rong, Weina Cao, Zhengchang Su, Guiping Zhao, Ranran Liu, Sumei Zhao, Ying Huang, Marinus F. W. te Pas, Changrong Ge, Junjing Jia

**Affiliations:** 1 Faculty of Animal Science and Technology, Yunnan Agricultural University, Kunming, 650201 Yunnan Province People’s Republic of China; 2 Yunnan Vocational and Technical College of Agriculture, Kunming, 650031 Yunnan Province People’s Republic of China; 3 College of Food Science, Yunnan Agricultural University, Kunming, 650201 Yunnan Province People’s Republic of China; 4 Yunnan University of Traditional Chinese Medical, Kunming, 650500 Yunnan Province People’s Republic of China; 5 Department of Bioinformatics and Genomics, College of Computing and Informatics, the University of North Carolina at Charlotte, Charlotte, NC 28223 USA; 6 Institute of Animal Sciences, Chinese Academy of Agricultural Sciences, Beijing, 100193 People’s Republic of China; 7 Wageningen Livestock Research, Wageningen UR, Wageningen, 238050 The Netherlands; 8 Visiting Professor Yunnan Agricultural University, Kunming, 650201 Yunnan Province People’s Republic of China

**Keywords:** Wuliangshang black-boned chickens, Melanogenesis, Muscle metabolite, Lysophospholipid, Transcriptomics, Metabolomics

## Abstract

**Background:**

Melanin is an important antioxidant in food and has been used in medicine and cosmetology. Chicken meat with high melanin content from black-boned chickens have been considered a high nutritious food with potential medicinal properties. The molecular mechanism of melanogenesis of skeletal muscle in black-boned chickens remain poorly understood. This study investigated the biological gene-metabolite associations regulating the muscle melanogenesis pathways in Wuliangshan black-boned chickens with two normal boned chicken breeds as control.

**Results:**

We identified 25 differentially expressed genes and 11 transcription factors in the melanogenesis pathways. High levels of the meat flavor compounds inosine monophosphate, hypoxanthine, lysophospholipid, hydroxyoctadecadienoic acid, and nicotinamide mononucleotide were found in Wuliangshan black-boned chickens.

**Conclusion:**

Integrative analysis of transcriptomics and metabolomics revealed the dual physiological functions of the PDZK1 gene, involved in pigmentation and/or melanogenesis and regulating the phospholipid signaling processes in muscle of black boned chickens.

**Supplementary Information:**

The online version contains supplementary material available at 10.1186/s12864-022-08388-w.

## Background

Melanins are pigment molecules that are endogenously synthesized by melanocytes found in skin, hair follicles, eyes, inner ear, bones, muscle, heart, and brain in human, animal and birds [1, 2]. Melanin is also considered an important antioxidant in food and has been used in medicine and cosmetology [3]. Chicken meat with high melanin content from black-boned chickens or Silky chickens have been considered a high nutritious meat with valuable meat quality and medicinal effects, including the enhancement of the human immune system [4], prevention of emaciation [5], treatment for diabetes [6], and female health conditions such as menoxenia and postpartum complications [7]. The molecular mechanism underlying these characteristics remains poorly understood.

Melanin-based pigmentation is under strong genetic control via the melanocortin pathway [1, 2]. Yu et al. [2] found 264 differentially expressed genes between black and white chicken breast muscles. Despite the identification of several melanogenesis related genes, the genetic factors involved in melanin pigmentation in chicken muscle are still poorly understood. The molecular mechanisms underlying the variation in the melanin pigmentation of chicken muscle associated with meat quality, nutrition or medicinal properties remain unclear. Integration of data on multiple levels including multi-omics technologies enables the characterization of genome-phenotype relationships and unravel biological molecular complexity [8]. Our purpose was to combine gene expression and metabolite abundance related to melanogenesis in skeletal muscle in chickens.

The Wuliangshan black-boned (WLS) chickens is a native chicken breed of Yunnan Province. With the exception of the feathers, it is characterized by an all-black body, including comb, break, skin, muscle, adipose tissue, internal organs, bone and periosteum. In our previous study WLS chickens showed high meat quality, such as good tasty, juiciness, tender, high intramuscular fat content and rich in flavor amino acid and polyunsaturated fatty acids compared to other local breeds or commercial broilers [9]. The molecular mechanism of meat quality characteristics associated with melanogenesis of skeletal muscle in WLS chickens remains unclear. Therefore, the present study integrated transcriptomics and metabolomics analyses to investigate the biological gene-metabolite associations. Skeletal muscle melanogenesis pathways were investigated to reveal the melanogenesis molecular mechanism of skeletal muscle associated with meat quality characteristics including health affecting metabolites. Two normal-boned chicken breeds including Cobb (CB) broilers and Chahua chickens (CH), a unselected local breed similar to WLS chickens in Yunnan Province, were used to eliminate genetic background effects.

## Results

### Transcriptome profiling in chicken breast muscle: differentially expressed genes and KEGG enrichment analysis

Sequencing obtained 235.57 Gb raw data with 1701.17 million sequences. After filtering, 233.02 Gb clean data with 1687.33 million sequences remained for subsequent analysis (Additional file 1: Table S2). Mapping to the jungle fowl genome *Gallus_gallus-5.0* showed that the mapped ratio for each sample was higher than 73% (Additional file 1: Table S2).

**Fig. 1:**
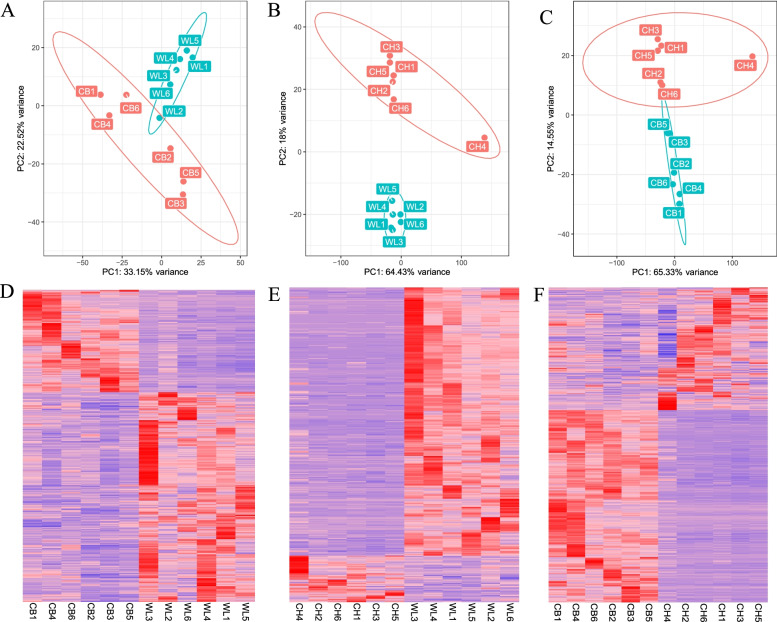
Principal component analysis (PCA) and heatmap of DEGs. **A** PCA of WLS black boned chickens (green) and Cobb broilers (red). **B** PCA of WLS black boned chickens (green) and Chahua chickens (red). **C** PCA of Chahua chickens (red) and Cobb broilers (green). **D** Heatmap of 750 differentially expressed genes (DEGs) with 504 genes with up-regulated expression (red) and 246 genes with down-regulated expression (blue) from WLS vs CB group. **E** Heatmap of 453 DEGs with 386 genes with up-regulated expression (red) and 67 genes with down-regulated expression (blue) from WLS vs CH group. **F** Heatmap of 936 DEGs with 365 genes with up-regulated expression (red) and 571 genes with down-regulated expression (blue) from WLS vs CB group

We obtained 750 DEGs with 504 up-regulated genes and 246 down-regulated genes from WLS vs CB group, 453 differentially expressed genes (DEGs) with 386 up-regulated genes and 67 down-regulated genes from WLS vs CH group, and 936 DEGs with 365 up-regulated genes and 571 down-regulated genes from CH vs CB group (Additional file 2). Figure 1 shows the PCA and heat maps of the WLS vs CB, WLS vs CH and CH vs CB analyses, respectively.

### Functional analysis of differentially expressed genes

**Fig. 2:**
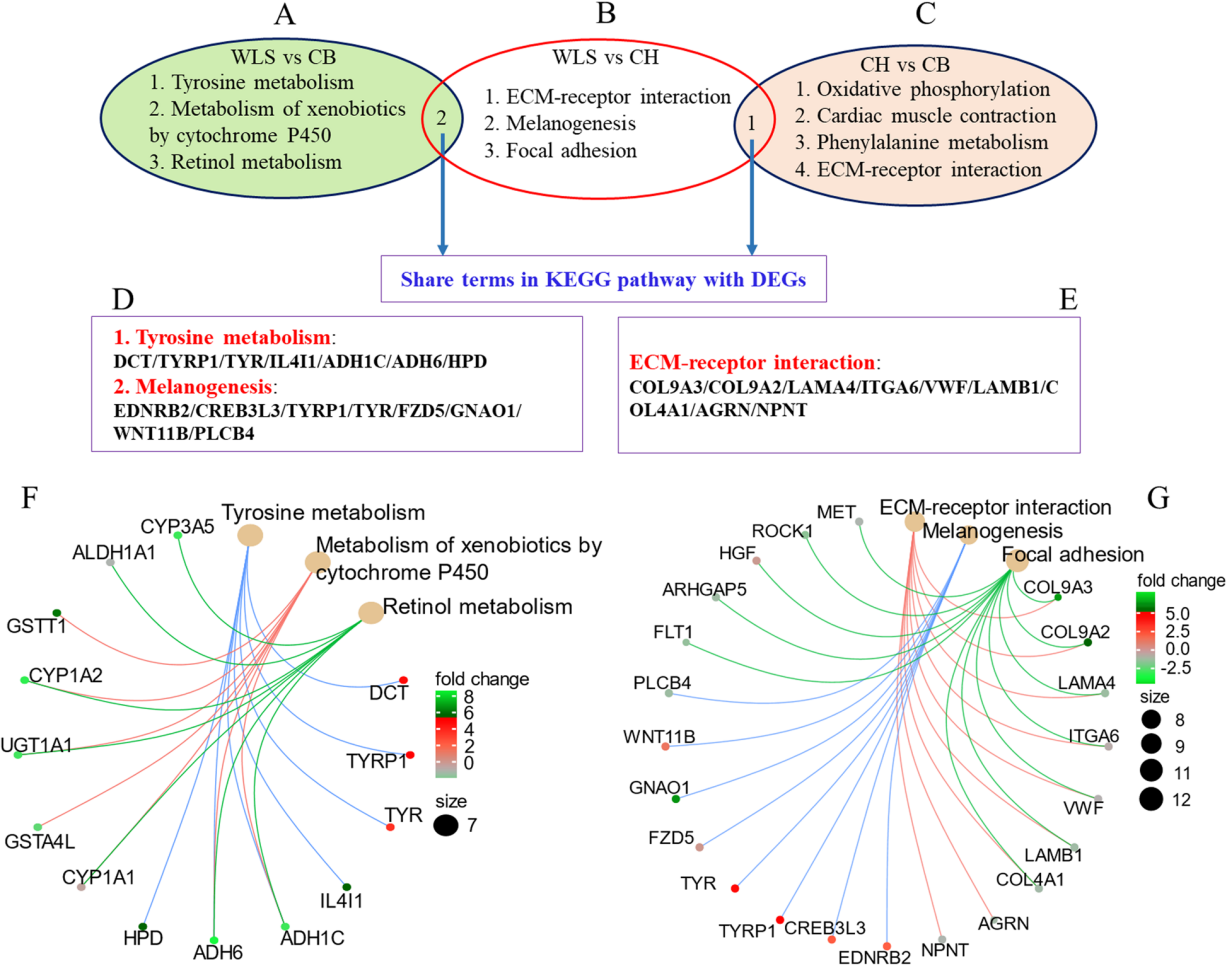
KEGG enrichment analysis for DEGs and Network plot of DEGs related to melanogenesis. **A** Three significantly enriched KEGG pathways from the DEGs in the WLS vs CB group. **B** Three significantly enriched KEGG pathways from the DEGs in the WLS vs CH group. **C** Four significantly enriched KEGG pathways from the DEGs in the CH vs CB group. **D** Two pathways including Tyrosine metabolism and Melanogenesis with shared terms in KEGG pathway with DEGs comparing the WLS vs CB and WLS vs CH groups, respectively. **E** The ECM-receptor interaction was a shared term in the KEGG pathway with DEGs comparing the WLS vs CH and CH vs CB groups, respectively. **F** Network plot of DEGs related to tyrosine metabolism (*DCT*, *TYRP1*, *TYR*, *IL4I1*, *ADH1C*, *ADH6* and *HPD*). **G** Network plot of DEGs related to the melanogenesis pathway (*EDNRB2*, *CREB3L3*, *TYRP1*, *TYR*, *FZD5*, *GNAO1*, *WNT11B* and *PLCB4*)

Figure 2 shows the significantly enriched pathways from the KEGG enrichment analysis. Three significantly enriched pathways were obtained from WLS vs CB (Fig. 2A) and WLS vs CH (Fig. 2B) while four significantly enriched pathways were obtained from CH vs CB (Fig. 2C). For the WLS vs CB (Fig. 2A) and the WLS vs CH comparisons (Fig. 2B), we found two significantly enriched KEGG pathways, the tyrosine metabolism and melanogenesis with a total of 15 differently expressed genes, which were associated with chicken muscle melanogenesis (Fig. 2D and E). Seven genes, including *DCT*, *TYRP1*, *TYR*, *IL4I1*, *ADH1C*, *ADH6* and *HPD* were involved in the pathway of tyrosine metabolism. Eight genes, including *EDNRB2*, *CREB3L3*, *TYRP1*, *TYR*, *FZD5*, *GNAO1*, *WNT11B* and *PLCB4* were involved in the pathway of melanogenesis in chickens. No significant KEGG pathway was enriched associated with tyrosine metabolism or melanogenesis from the CH vs CB comparison (Fig. 2C).

Furthermore, we obtained a total of 24 DEGs associated with the tyrosine metabolism and melanogenesis pathways and eleven transcription factors by comparative analyses in different species including human, mammals and fish (Table 1). Six DEGs including *GPR143*, *ALDH1A1*, *MC5R*, *CDKN2B*, *PDZK1* and *PDZK1IP1*, were reported for the first time in chicken muscle associated with tyrosine metabolism and melanogenesis. We found that WLS black boned chickens showed significantly higher mRNA expression of transcription factors genes including *TFAP2B*, *TFAP2A*, *TCF21* and *ELF3* compared to the other chicken breeds. The four transcription factors genes might regulate the tyrosine metabolism and melanogenesis in chicken muscle.

**Table 1:** Gene expressions associated with melanogenesis

Gene	Symbol	WLS vs CB (Fold change)		WLS vs CH (Fold change)	
Full name		RNA-Seq	qPCR	RNA-Seq	qPCR
premelanosome protein	PMEL	8.98	9.24	8.35	8.96
tyrosinase related protein 1	TYRP1	8.27	10.54	7.41	6.54
tyrosinase	TYR	7.05	8.23	7.15	5.43
melan-A	MLANA	6.04	7.23	6.84	7.34
melanophilin	MLPH	5.33	5.62	8.28	9.23
premelanosome protein 17	Pmel17	5.95	4.89	5.58	4.98
G protein-coupled receptor 143	GPR143	6.36	7.23	2.91	3.43
aldehyde dehydrogenase 1 family member A1	ALDH1A1	3.28	4.12	1.69	1.89
endothelin 3	EDN3	2.27	2.87	1.95	2.45
endothelin receptor B subtype 2	EDNRB2	2.15	3.23	4.62	5.62
cyclin dependent kinase inhibitor 2B	CDKN2B	1.74	3.23	3.39	4.56
PDZ domain containing 1	PDZK1	2.14	4.12	2.54	3.23
PDZK1 interacting protein 1	PDZK1IP1	1.45	2.43	2.54	2.98
dopachrome tautomerase	DCT	7.99	8.35	2.13	3.12
cAMP responsive element binding protein 3 like 3	CREB3L3	NS	3.21	4.74	3.43
wingless-type MMTV integration site family, member 11b	WNT11B	NS	2.12	3.87	2.98
frizzled class receptor 5	FZD5	NS	2.43	2.63	3.23
melanocortin 5 receptor	MC5R	NS	2.16	2.26	2.98
alcohol dehydrogenase 1C (class I), gamma polypeptide	ADH1C	1.59	1.98	NS	2.01
MET proto-oncogene, receptor tyrosine kinase	MET	NS	3.32	1.57	1.79
phospholipase C beta 4	PLCB4	NS	2.12	1.28	2.01
alcohol dehydrogenase 6 (class V)	ADH6	1.05	1.83	NS	2.12
G protein subunit alpha o1	GNAO1	NS	−2.65	−3.21	−2.32
interleukin 4 induced 1	IL4I1	−1.61	− 2.32	NS	− 1.98
4-hydroxyphenylpyruvate dioxygenase	HPD	−1.58	−2.76	NS	−2.32
Transcription factors					
forkhead box A1	FOXA1	2.43	4.32	6.06	5.35
transcription factor AP-2 beta	TFAP2B	5.26	5.43	4.35	4.67
transcription factor AP-2 alpha	TFAP2A	3.46	5.12	5.55	6.45
transcription factor 21	TCF21	1.31	2.32	5.04	4.34
E74 like ETS transcription factor 3	ELF3	1.52	2.32	4.74	3.76
SRY-box 10	SOX10	1.95	2.32	NS	2.42
activating transcription factor 3	ATF3	3.04	4.32	NS	4.87
runt-related transcription factor 1	RUNX1	−2.54	−1.98	NS	−2.43
JunD proto-oncogene, AP-1 transcription factor subunit	JUND	1.77	2.22	NS	2.54
MAF bZIP transcription factor A	MAFA	1.6	2.01	NS	1.98
SAM pointed domain containing ETS transcription factor	SPDEF	NS	2.31	4.33	3.21

*NS* Not significant different

### Metabolomics analysis for metabolite profiling in breast muscle

**Fig. 3:**
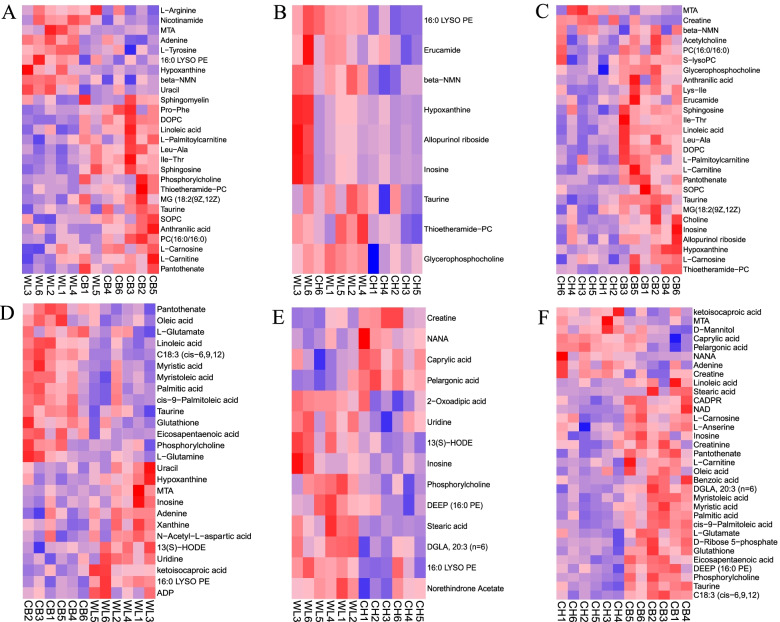
Heatmaps of differential abundant metabolites (significantly differential metabolites (SDMs) or differential metabolites (DMs)) in each individual animal from three groups in positive or negative ion mode analysis**.** Based on the model of OPLS DA, we defined VIP > 1 and *P* < 0.05 as standard to measure SDMs, defined VIP > 1 and 0.05 < *P* < 0.1 as standard to DMs. **A** Heatmaps of 17 SDMs and 10 DMs with 9 high abundance (red) or 18 low abundance (blue) from WLS vs CB group in positive ion mode. **B** Heatmaps of 5 SDMs and 4 DMs with 9 high abundance (red) from WLS vs CH group in positive ion mode. **C** Heatmaps of 19 SDMs and 8 DMs with 3 high abundance (red) and 24 low abundance (blue) from CH vs CB group in positive ion mode. **D** Heatmaps of 16 SDMs and 8 DMs with 12 high abundance (red) or 15 low abundance (blue) from WLS vs CB group in negative ion mode. **E** Heatmaps of 12 SDMs and 2 DMs with 10 high abundance (red) or 4 low abundance (blue) from WLS vs CH group in negative ion mode. **F** Heatmaps of 24 SDMs and 8 DMs with 8 high abundance (red) and 24 low abundance (blue) from CH vs CB group in positive ion mode

Based on the model of OPLS DA, we defined VIP > 1 and *P* < 0.05 as standard to measure significantly differential metabolites (SDM), and VIP > 1 and 0.05 < *P* < 0.1 as standard to measure differential metabolites (DM). Figure 3 A, B and C shows the heat maps of SDM and DMs for positive ion mode, ands Fig. 3 D, E and C for negative ion mode in each of the comparisons.

**Fig. 4:**
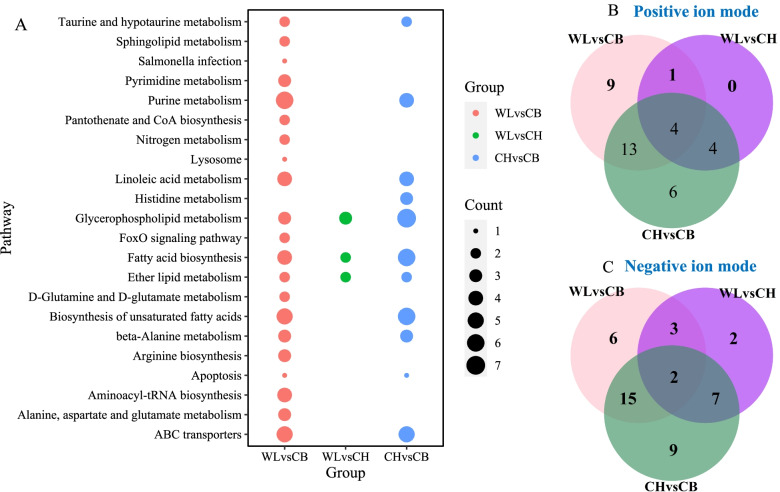
KEGG enrichment analysis for SDMs or DMs and shared terms of SDMs or DMs from three comparisons: WLS vs CB, WLS vs CH, and CH vs CB. **A** Significantly enriched KEGG pathways, **B** Venn diagraph showing common metabolites in positive ion mode. **C** Venn diagraph showing common metabolites in negative ion mode

In positive ion mode (Additional file 3: Table S4–6), we obtained 17 SDMs and 10 DMs with 9 high and 18 low abundances, respectively, for the WLS vs CB comparison (Fig. 3A), 5 SDMs and 4 DMs with 9 high abundances, respectively, for the WLS vs CH comparison (Fig. 3B), 19 SDMs and 8 DMs with 3 high and 24 low abundances, respectively, for the CH vs CB comparison (Fig. 3C). In negative ion mode (Additional file 3: Table S7–9), we obtained 16 SDMs and 9 DMs with 12 high and 15 low abundances, respectively, for the WLS vs CB comparison (Fig. 3D), 12 SDMs and 2 DMs with 10 high or 4 low abundances, respectively, for the WLS vs CH comparison (Fig. 3E), 26 SDMs and 8 DMs with 8 high or 24 low abundances, respectively, for the CH vs CB comparison (Fig. 3F). Twenty-two, three and eleven KEGG pathways for SDM or DMs were enriched respectively for WLS vs CB, WLS vs CH and CH vs CB groups (Fig. 4A). We found three pathways including glycerophospholipid metabolism, Fatty acid biosynthesis and Ether lipid metabolism common for all three comparisons.

Taurine, hypoxanthine, 1-Palmitoyl-2-hydroxy-sn-glycero-3-phosphoethanolamine (16:0 LYSO PE) and Thioetheramide-PC were the common metabolites found in positive ion mode (Fig. 4B). Inosine and phosphorylcholine were the common metabolites found in negative ion mode (Fig. 4C). WLS chickens showed significantly higher abundance of 16:0 LYSO PE, inosine and its metabolite hypoxanthine in muscle compared to the CB and CH chickens both from positive and negative ion modes. WLS chickens showed significantly higher abundance of 13(S)-HODE in muscle compared to the CB and CH chickens in positive ion mode. WLS chickens had the highest abundance of beta-Nicotinamide D-ribonucleotide (β-NMN) in positive mode and S-Methyl-5′-thioadenosine (MTA) in both ion modes in muscle while CB broilers showed the lowest abundance of β-NMN and MTA in muscle. In contrast, CB broilers had the highest abundance of thioetheramide-PC (positive mode) and phosphorylcholine (both modes) while CH chickens showed the lowest abundance of thioetheramide-PC and phosphorylcholine in muscle. Thus, compared to CB broilers, local chicken breeds showed higher abundance of β-NMN and MTA in muscle but low abundance of thioetheramide-PC and phosphorylcholine in muscle (Additional file 3: Table S4–9).

### Integrated transcriptome and metabolome analysis

**Fig. 5:**
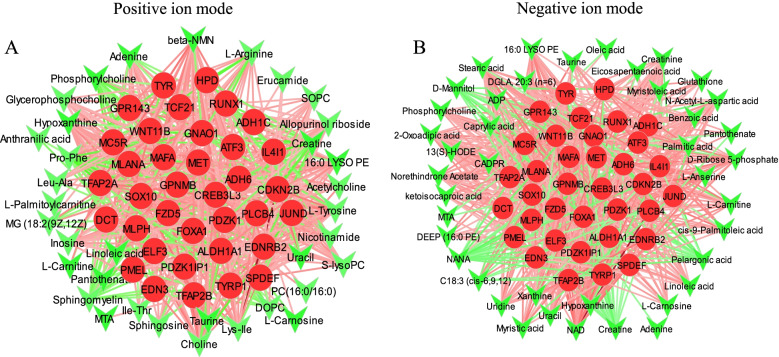
Correlation network of selected differentially expressed genes and metabolites. **A** Correlation network of differentially expressed genes and metabolites in positive ion mode, **B** Correlation network of differentially expressed genes and metabolites in negative ion mode

We identified a relationship between gene expression and the abundance of metabolites from correlation analysis of DEGs, SDM and DMs. Additional file 4 (Tables positive and negative ion modes) shows the correlation coefficient between DEGs and SDM/DMs. Figure 4 shows the correlation network of DEGs associated with melanogenesis and metabolites. Figure 5A (and Additional file 4: Table S1-Positive ion mode) shows significant positive correlations between the expressions of DEGs associated with melanogenesis and abundance of muscle metabolites including 16:0 LYSO PE, Adenine, β-NMN, Inosine, Hypoxanthine and L-Tyrosine. In contrast, abundance of the muscle metabolites DOPC, phosphorylcholine, Pro-Phe and sphingomyelin were significantly negative correlated with the expressions of DEGs associated with melanogenesis. Figure 5B (and Additional file 4: Table S2-Negative ion mode) shows that the abundance of muscle metabolites including 13(S)-HODE, 16:0 LYSO PE, hypoxanthine, ketoisocaproic acid, norethindrone acetate, stearic acid and uridine in negative ion mode were significantly positive correlated with the expressions of DEGs associated with melanogenesis while contrary cases were observed for C18:3 (cis-6,9,12), creatine and NANA (*P* < 0.05 or *P* < 0.01). The abundance of muscle 16:0 LYSO PE was significantly positive correlated with expressions of DEGs associated with melanogenesis both in positive and negative ion modes.

## Discussion

### Melanogenesis related gene expression in breast muscle

**Fig. 6:**
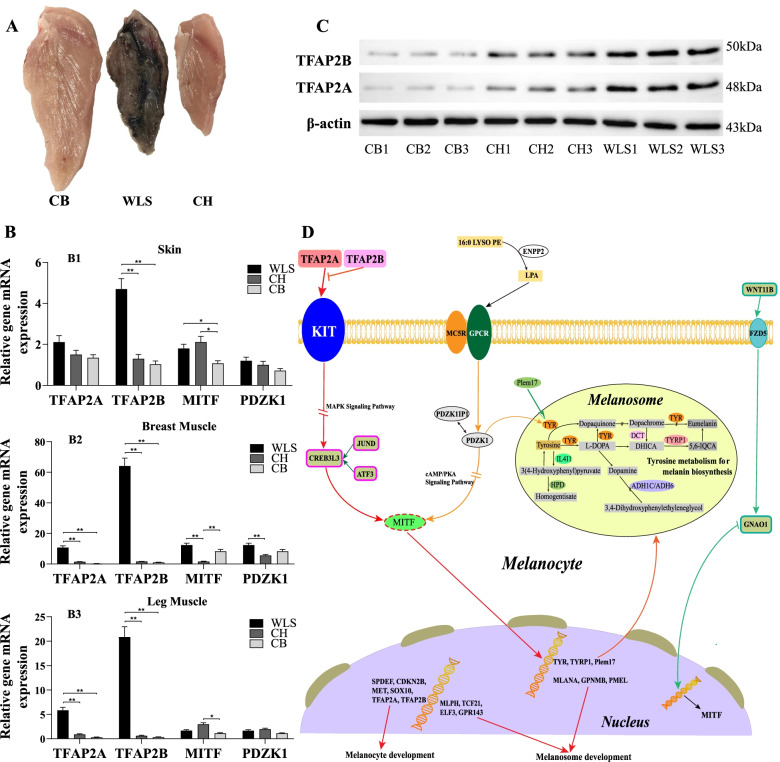
Regulatory network of melanin deposition in chicken breast muscle. **A** Breast muscle image from three chicken breeds. (**B**1–3) Relative expression of *TFAP2A*, *TFAP2B*, *MITF* and *PDZK1* gene mRNA from skin (**B**1), breast muscle (**B**2) and leg muscle (**B**3) at 1 day of age of three chicken breeds. **C** Western-blot analysis for protein expression levels of TFAP2A and TFAP2B. For full length blot information see additional file 5. **D** Melanin synthesized within melanosomes of melanocytes by a series of reactions that are catalyzed by specific melanogenic enzymes and transcriptional factors in muscle of chickens. Production of these enzymes is driven by the *MITF* transcription factor whose activity is regulated by a number of signaling pathways including MAPK (red), cAMP/ PKA (orange) and WNT (green). These signaling pathways are activated upstream by receptors such as *KIT*, *MC5R* and *FZD5*. *TFAP2A* and *TFAP2B* could regulate the *KIT* gene expression, which is the important gene affecting upstream of MAPK signaling pathways in melanin deposition. Similarly, the *MC5R* gene could regulate *MITF* gene through *CREB3L3*, and the *GPCG* gene could regulate *MITF* gene through *PDZK1*. As the target gene of *MITF*, *TYR*, *TYRP1* and *Plem17* directly participate in the tyrosine metabolism signaling pathway with the *DCT*, *IL4I1*, *HPD*, *ADH1C* and *ADH6* genes. *MLANA*, *GPNMB* and *PMEL* could affect the melanosome development. Furthermore, we proposed that lipid molecule 16:0 LYSO PE could be converted into LPA under *EPNN2*, and then LPA could activate upstream receptors *GPCR* to regulate the *PDZK1* gene expression involved in tyrosine metabolism and the melanogenesis signaling pathways

Melanogenesis is the production of the melanin pigments by melanocytes [10]. Melanocytes can be identified by the expression of melanocyte-specific markers such as tyrosinase (TYR), tyrosinase-related protein 1 (TYRP1), DOPAchrome tautomerase (DOT) or tyrosinase-related protein-2 (TYRP2), PMEL (premelanosome protein 17, Pmel17), MLANA (melan-A), melanoma antigen recognized by T cells 1 (MART-1), and microphthalmia-associated transcription factor (MITF) [11]. In the present study, WLS black-boned chickens showed higher gene expression of melanocyte-specific markers *TYR*, *TYRP1*, *DOT*, *PMEL 17* and *MLANA* in breast muscle compared to two chicken breeds with white breast muscle. Melanoblasts, the precursor cells of melanocytes, are unpigmented cells that migrate to various regions of the body and develop into melanocytes. Melanoblasts development into melanocytes was found predominantly in the myolemma and myocytes in chicken breast muscle (Fig. 6A). We suggest that melanoblasts might migrate to bone, marrow, myolemma and myocytes and then develop to melanocytes in black boned chickens.

WLS black-boned chicken showed significantly higher gene expression for five melanocyte-specific markers (*TYR*, *TYRP1*, *DOT*, *PMEL 17* and *MLANA*) and fifteen genes associated with the melanogenesis pathway compared to the two white muscled chicken breeds. Our observation is consistent with previous reports in mammals and chicken [2, 12] that these genes are critical enzymes and structural proteins in the melanogenesis pathway. Six of these genes were identified for the first time in chicken muscle.

Melanin production is initiated and regulated by a number of signaling systems and transcription factors including the tyrosine kinase receptor KIT, its ligand SCF, as well as *MITF* [13]. In vertebrates and mammals *MITF* is the only member of the microphthalmia family of transcription factors known to be essential for melanocyte development [1]. We did not find a significant difference of *MITF* mRNA expression in breast muscle between the WLS black-boned chickens and two white muscled chicken breeds. Our observation is consistent with previous reports in chicken that no significant different expression of *MITF* mRNA in breast muscle was measured between black-boned chicken and normal boned chickens [12]. Similarly, no significant different expression was measured in the skin between the black- and white-skinned chickens [14]. Yu et al. [2] showed that *MITF* played an important role regulating the plumage melanogenesis in black-boned chicken. We observed a different transcriptional regulatory mechanism for melanogenesis in muscle tissue of chickens. Different expressions of *MITF* mRNA were determined in skin, breast muscle and leg muscle tissues from 1 day aged chickens. Only in breast muscle WLS black boned chickens showed higher expression than in the two white muscle chicken breeds. Our findings suggest that *MITF* might not play a central role in the melanogenesis of chicken muscle.

WLS black-boned chickens showed significantly higher expression of the transcription factor AP-2 (*TFAP2B* and *TFAP2A*) in breast muscle at transcriptome, qPCR and protein Western-blot analysis at chickens aged 90 days related to melanogenesis related genes, compared to the two white muscled chicken breeds. *TFAP2B* transcriptionally regulates the development of cornea cells and may be involved in pigment production in the eye. These results may suggest that *TFAP2B* and *TFAP2A* transcriptionally regulate melanocyte-specific marker genes and therefore may be important transcriptional regulators of melanoblasts development in chicken muscle. These findings suggest that *TFAP2A* and *TFAP2B* act as central transcriptional factors promoting melanoblasts differentiation in parallel with *MITF*, and might play an important role in melanogenesis of chicken muscle. *TFAP2A* and *TFAP2B* might be important candidate genes in molecular selection of black-boned chickens.

### Muscle metabolites potentially related to meat quality

In our previous study we showed that WLS chickens showed high meat quality, such as good tasty, juiciness, tender, high intramuscular fat content and rich in flavor amino acid and polyunsaturated fatty acids compared to other local breeds or commercial broilers [9]. In the present study, two local Chinese native breeds showed high levels of IMP and hypoxanthine in breast muscle. Especially the WLS black-boned chickens showed higher levels of IMP and hypoxanthine in breast muscle than the other two white muscle chicken breeds. This is consistent with a previous study that black-boned chickens showed higher level of IMP in muscle associated with excellent flavor and taste in meat [15]. IMP is a flavor enhancer that is 50 times more potent than monosodium glutamate and has been used internationally as an important index for measuring meat flavor [16]. Hypoxanthine is produced by the degradation of IMP during postmortem aging of meat. The hypoxanthine and IMP contribute an umami taste, favored by many consumers.

WLS black-boned chickens showed higher levels of L-arginine and L-tyrosine than that of in broiler (WLS vs CB) while CB broiler showed the highest levels of leucine-alanine, isoleucine-threonine, proline-phenylalanine in breast muscle among the three chicken breeds. Our results suggest that composition of free amino acid in chicken breast muscle that contribute to meat aroma and flavor might be breed-specific. Nitrogen compounds such as free amino acid or small peptides contribute to meat flavor formation [17]. Ichimura et al. [18] showed that contents of free amino acids, small peptides, INO and hypoxanthine increased, while those of adenosine monophosphate and IMP decreased during postmortem aging.

### Muscle metabolites potential nutritional and medicinal properties

Several of the metabolites may have important potential nutritional and medical properties. Because of the significant differences between black muscled and white muscled meat characteristics this may be valuable for human consumption and health.

#### 1-Palmitoyl-2-hydroxy-sn-glycero-3-phosphoethanolamine (16:0 LYSO PE)

WLS black boned chicken showed significantly higher metabolite levels of 16:0 LYSO PE in breast muscle than that of in two chicken breeds with white muscle, and may be involved in the melanogenesis of skeletal muscle in chicken.

#### Hydroxyoctadecadienoic acid (13-HODE)

WLS black-boned chickens showed higher levels of linoleic acid oxidation product, 13-HODE and lower levels of phosphatidylcholine in breast muscle compared with the two white muscled chicken breeds. Fang et al. [19] indicated that 13- HODE has vasoactive properties, was rapidly taken up by bovine aortic endothelial cells. 13-HODE prevents cell adhesion to endothelial cells and can inhibit cancer metastasis. 13-HODE is a stable oxidation products and have been linked to pathological conditions including atherosclerosis, diabetes, Alzheimer’s disease, non-alcoholic steatohepatitis, psoriasis, chronic inflammation, obesity, and cancer [20, 21]. 13-HODE, is abundantly produced during vascular activation [22]. 13-HODE induced vascular activation and could play a role in regulating endothelial barrier integrity during inflammation [22, 23]. WLS black-boned chicken showed high level of 13-HODE in breast muscle that might play a role contributing to these medicinal properties, including enhancement of the human immune system [4], female health conditions including menoxenia and postpartum complications [7].

#### Beta-nicotinamide D-ribonucleotide (β-NMN)

WLS black boned chicken showed higher β-NMN levels in breast muscle compared to the two white muscled chicken breeds. Mostly mediated by its involvement in NAD+ biosynthesis, the pharmacological activities of β-NMN include its role in cellular biochemical functions, cardio protection, diabetes, Alzheimer’s disease, and complications associated with obesity, and to counteract age-associated pathologies associated with a decline in tissue NAD+ levels [24]. Administration of β-NMN can compensate for the age-related NAD+ deficiency. Thus black-boned chickens may be considered as high nutritious food with medicinal properties. WLS black-bone chicken may be an important genetic resource for health poultry production due to its abundant NMN content as anti-aging agent for food.

### Integrative analysis of transcriptomics and metabolomics to reveal the muscle pathway of melanogenesis

Correlation analysis showed that there were significant positive correlations between the muscle 16:0 LYSO PE metabolite level and five melanocyte-specific marker genes (*TYR*, *TYRP1*, *DOT*, *PMEL 17* and *MLANA*) and fifteen genes associated with the melanogenesis pathway. Integrating the expression of the melanocyte-specific marker genes and metabolite abundance enabled to postulate a model summarizing the melanogenesis in WLS muscle (Fig. 6). Lysophospholipid (LPL) is a metabolite related to 16:0 LYSO PE. Our results suggest that muscle LPL might played an important role involved in the melanogenesis pathway in muscle. We suggest that LPL might stimulate melanocytes migration or melanin deposition in skeletal muscle in black boned chickens. It implicates that black boned chicken possess a specific molecular mechanism of melanogenesis pathway regulating LPL metabolic signaling in muscle.

LPL as intermediary phospholipid metabolite plays an important role in the phospholipid metabolism signaling pathway. The LPLs signals through G-protein coupled receptors (*GPCRs*), which control phospholipid signaling processes within the target cell. Phospholipase C-β (PLC-β) is a key molecule in the G protein-coupled receptor (GPCR)-mediated phospholipid signaling. Four PLC-β subtypes have different physiological functions despite their similar structures [25]. PLC-β subtypes possess different PDZ-binding motifs, and they have the potential to interact with different PDZ proteins. PDZK1 is a PDZ protein interacting with the somatostatin receptors (SSTRs). Through these interactions, PDZK1 assembles as a ternary complex with PLC-β3 regulating the phospholipid signaling processes [26]. We suggest that LPL signaling through G-protein coupled receptors (GPCRs) control phospholipid signaling processes within the cell via ternary complex of PDZK1, SSTRs and PLC-β3 in muscle tissue of black boned chicken. WLS black boned chicken showed significantly higher LPL in breast muscle than the two white muscled chicken breeds, and this is associated with significant high *PDZK1* mRNA and protein expression. Previous studies showed that overexpression of PDZK1 increased tyrosinase expression. *PDZK1* knockdown reduced estrogen-induced tyrosinase expression. Thus, estrogen is involved in regulating melanogenesis through *PDZK1* in melisma cell [26]. We therefore propose that the biological function of the *PDZK1* gene is both involved in melanogenesis and regulating the phospholipid signaling processes in muscle of black boned chickens (Fig. 6D).

## Conclusion

We propose the melanogenesis pathway in black boned chicken muscle derived from integrated transcriptomics and metabolomics results of black and white muscled chicken breeds. Significant high contents of muscle valuable metabolites including 13-HODE, NMN and LPL in black-boned chickens suggested important health related and medicinal properties.

## Materials and methods

### Animal experimentation ethical statement

All procedures conducted with the chickens were approved by the Yunnan Agricultural University Animal Care and Use Committee (approval ID: YAUACUC01). Animal use and care were in accordance with the Guide for the Care and Use of Laboratory Animals published by the US National Research Council.

### Chicken, diet and housing

One-day-old WLS and CH chickens were purchased from the Yunnan Agricultural University Chicken Farm. One-day-old CB chickens were purchased from the Kunming Zhengda Group (Kunming, Yunnan, P. R. China).

A total of 90 chickens of 1 d of age (30 from each chicken breed) were reared under standard conditions on a starter diet (Period I: 20.6% CP and 12.8 MJ /kg ME) to 30 d of age. From 30 d of age onward, chickens were fed a regular diet (Period II: 18.4% CP and 12.5 MJ /kg ME) to 90 days (week 12). Diet content was consistent with the formulation to meet NRC 1994 and Chinese Chicken Feeding Standard recommendations. The compositions of diets and housing details are shown in Additional file 1: Table S3. All chickens were sacrificed at week 12.

### Slaughter procedure and sample collecting

Feed was withdrawn 16 h and water 12 h before slaughter. The body weight (BW) of the chickens was measured at 90 d of age in the morning. Chickens were slaughtered by cervical dislocation in accordance with the National Experimental Animal Slaughter Standard of China. Six breast muscle samples (100 mg) from each chicken breed, were collected and placed in sterile tubes (RNase-free), which were immediately snap frozen in liquid nitrogen prior to storage at − 80 °C for subsequent analysis of the transcriptome or gene expression in Real-time PCR. From the same chickens 600 mg samples of breast muscle were collected and placed in sterile tubes (RNase-free), and immediately snap frozen under liquid nitrogen for subsequent analysis of the metabolome. All samples were stored at − 80 °C until processing.

### Sample preparation for transcriptome analysis

#### RNA preparation

Total RNA of breast muscle samples was isolated using the TaKaRa MiniREST Universal RNA Extraction Kit (TaKaRa Biotechnology Co., Ltd., Dalian, P.R. China) according to the manufacturer’s protocol. The purity was detected by the Nanodrop 2000 spectrophotometer (Nanodrop, Wilmington, DE, USA). The OD260/OD280 ratio of all samples was 1.8 to 2.0. The concentration of total RNA was determined using the Qubit 2.0 Fluorometer (Thermo Fisher Scientific Inc., MA, USA), and the integrity was detected using the Angilent 2100 Bioanalyzer (Agilent Technologies, Inc., Santa Clara, CA, USA). The total RNA samples were stored at − 80 °C until further use.

#### cDNA library preparation and sequencing using RNA-Seq

To enrich the mRNA, two rounds of hybridization to oligo (dT) beads were performed on 7 μg total RNA of each sample. Ribosomal RNA contamination was analyzed using an RNA picochip with a BioAnalyzer (Agilent Technologies, Inc., Santa Clara, CA, USA). The mRNA generated was used to establish cDNA libraries using an RNA-seq sample preparation kit (Illumina, San Diego, CA, USA) as described by Zhang et al. [27]. The cDNA libraries were sequenced separately using an Illumina HiSeq 2500 with a 100-bp pair-end read length.

### Transcriptome analysis

#### Raw data quantification

Raw sequence data were obtained and checked including per base sequence quality, per sequence quality score, per base N count and overrepresented sequences using software FastQC (Version 0.11.5, https://github.com/s-andrews/FastQC). High quality raw data was filtered by software Trim Galore (Version 0.4.4, https://github.com/FelixKrueger/TrimGalore). Reads with adaptor, repetitive or low-quality, and the unknown base (N) greater than 5% were removed.

#### Reads alignment

The *Gallus gallus* genome (ftp://ftp.ensembl.org/pub/release-94/fasta/gallus_gallus/dna/Gallus_gallus.Gallus_gallus-5.0.dna.toplevel.fa.gz) from the Ensembl database was selected to map with clean data using software HISAT2 (Version 2.0.4, https://github.com/infphilo/hisat2) to obtain SAM files containing alignment. Samtools (Version 1.5, https://github.com/samtools/samtools) was used to transfer SAM files to BAM files for further analysis.

#### Gene expression analysis

DEseq2 (https://github.com/mikelove/DESeq2) was used to analyze gene expression. The padj (FDR) represented the significance of differentially expressed gene, and log_2_FoldChange (log_2_FC) represented relative gene expression level. We defined log_2_FC ≥ 1 and FDR < 0.05 to finally obtain differentially expressed genes (DEGs) [28]. In order to analyze the differentially expressed genes we needed to normalize the gene count matrix. We calculated the normalization factors with specific values. For specific details see Supplementary file 1, transcriptomic data normalization.

#### KEGG pathway enrichment analysis

The KEGG (Kyoto Encyclopedia of Genes and Genomes) database was used for enrichment analysis of pathways of differentially expressed genes, and to investigate the possible biological functions [29].

### Verification of the RNA-Seq results

qPCR analysis has been performed to validate 36 differentially expressed genes (DEGs) in tyrosine metabolism and melanogenesis pathways using the Bio-Rad CFX96 real-time PCR platform (Bio-Rad Laboratories. lnc, America). The same animals as for transcriptome and metabolome analyses were used. Total RNAs were extracted from breast muscle, leg muscle and skin tissue of 1 day aged chickens [30]. Additional file 1: Table S1 describes the gene primers (Invitrogen Biotechnology Co., Ltd., Shanghai, China). The 2^−ΔΔCt^ method was used to analyze mRNA abundance. All samples were analyzed in triplicate and the average values of these measurements were used to calculate the expression of mRNA [31].

### Western-blot analysis

Western blots were performed with the antibodies anti-TFAP2A and anti-TFAP2B from Novus Biologicals lnc. (Littleton, USA). Breast muscle samples were taken from the same individuals as used for transcriptome and metabolite analyses. Muscle total protein extraction was performed with RIPA lysis buffer supplemented with PMSF (Solaibao Technology Co. LTD, Beijing, China), and total protein concentration was determined by BCA Protein AssayKit (Biyuntian Biotechnology Co., LTD, Shanghai, China). The protein extracts were separated on reducing SDS 10% polyacrylamide gel electrophoresis, and then transferred onto a polyvinylidene fluoride membrane with 300 mA for 2 h. For antibody hybridization, the PVDF membrane was sealed with TBST solution containing 5% skimmed milk at room temperature for 1.5 h or 4 °C overnight. The membrane was incubated with polyclonal primary anti-TFAP2A and anti-TFAP2B diluted 1:1000 in TBST buffer containing 5% skimmed milk for 2.5 h. After a wash step, the membranes were finally incubated for 1.5 h with horseradish peroxidase conjugated with anti-mouse IgG diluted 1:5000 in PBS buffer, and the immunoreactions were detected by AI600 chemiluminescence imaging system (General Electric Company, Boston, USA).

### Metabolomic and metabolite analyses

#### Sample preparation for metabolome analysis

Global metabolite profiles were determined from muscle tissue of the same chickens as transcriptome analysis. Metabolomic profiling analysis was performed by Metabolon as previously described [8]. In short: 800 μl precooled methyl alcohol/acetonitrile (1:1, v/v) was added to the homogenized samples and vortexed. The samples were ultra-sonicated in a cold water bath and then placed at − 20 °C for 1 h. The mixtures were centrifuged for 15 min (13,000 rpm, 4 °C), and the supernatants were collected and freeze dried.

#### LC-MS/MS analysis for metabolome analysis

Sample separation was performed using an UHPLC (1290 Infinity LC, Agilent Technologies) HILIC and RPLC. The column temperature was 25 °C and flow rate was 300 μl/min with 2 μl of each sample loaded. The mobile phase of chromatography composed buffer A (Water + 25 mM ammonium acetate + 25 mM ammonium hydroxide) and buffer B (Acetonitrile). The gradient was (1) 95% buffer B for 1 min; (2) thereafter linearly reduced to 65% during 13 min (3) then reduced to 40% the following 2 min; (4) maintained at 40% for 2 min; (5) increased to 95% in 0.1 min; (6) kept at 95% for 5 min. To avoid the influence caused by signal fluctuation, samples were analyzed randomly. QC samples were inserted into the analysis queue to evaluate the system stability and data reliability during the whole experimental process.

Sample analyses were performed using an UHPLC coupled to a quadrupole time-of-flight (AB SCIEX TripleTOF 5600) in both ESI positive and negative modes. The ESI source conditions following HILIC separation were: Ion Source Gas1 (Gas1) as 60, Ion Source Gas2 (Gas2) as 60, curtain gas as 30, source temperature: 600 °C, Ion Spray Voltage Floating 5500 V in positive mode, and − 5500 V in negative mode. In MS only acquisition, the instrument was set to anm/z range 60–1000 Da, product ion scan m/z range 25–1000 Da, TOF MS scan accumulation time 0.20 s/spectra, product ion scan accumulation time 0.05 s/spectra. The product ion scan is acquired using information dependent acquisition (IDA) with high sensitivity mode selected. The collision energy was fixed at 35 V ± 15 eV. Declustering potential was set as ±60 V. IDA was set to exclude isotopes within 4 Da, Candidate ions to monitor per cycle: 6.

### Data processing and statistical analysis

The generated sequence reads were filtered and the reads that contained numerous interspersed Ns in their sequences and comparatively short reads (< 17 bp) were removed. The remaining sequence reads were subsequently analyzed using the CLC Genomics Workbench 4. After mapping, the unique gene reads for all of the 17,108 annotated chicken genes in the database from the six libraries were combined and analyzed using the DESeq R package. The DEGs between each two breeds (WLS vs CB, CB vs CH, and WLS vs CH respectively) were identified at combined cut-offs with *P* < 0.05 and a fold-change > 2. Functional annotations for the DEGs and the statistical analysis of the significantly represented functional categories were performed using DAVID [32] (https://david.ncifcrf.gov/). Significance was set at a fold-enrichment > 2, *P* < 0.05, and a false discovery rate (FDR) < 20% for the pathway analysis.

For the metabolome analysis, the raw MS data were converted to MzXML files using ProteoWizard MS Convert and processed using XCMS for feature detection, retention time correction and alignment. The metabolites were identified by accuracy mass (< 25 ppm) and LC-MS/MS data, which were matched with the standards database. In the extracted ion features, only the variables having nonzero measurement values in more than 1/3 of the samples were kept. SIMCA-P 14.1 (Umetrics, Umea, Sweden) was used for principal component analysis (PCA), partial least-squares-discriminant analysis (PLS-DA) and orthogonal partial least-squares-discriminant analysis (OPLS-DA), after Pareto-scaling. Single dimensional statistical analysis includes Student’s t test and fold change. The volcano plot was obtained by R software.

The workflow: we first checked the integrity of the metabolomics data, deleted extreme values, and supplemented the missing values where possible. Then, the total peak area of the data was normalized by adding the total peak areas of all metabolites and dividing the peak area of each metabolite by the total area to get the content of each metabolite). Pareto-scaling was performed on the data used SIMCA-P (Sartorius GmbH, Goettingen, Germany). Partial Least Squares Discrimination Analysis (PLS-DA), a supervised statistical method, was used to analyse the metabolomics data. The partial least squares regression was used to establish the relationship model between the metabolite abundance level and the sample to predict the sample category. At the same time, Variable Importance for the Projection (VIP) was calculated to measure the influence intensity and explanatory ability of each metabolite expression pattern on the classification and discrimination of each group of samples, which could assist the screening of marker metabolites. In the PLS-DA model, the model evaluation parameters (R2Y, Q2) obtained through 7-fold cross-validation, and the closer R2Y and Q2 were to 1, the more stable and reliable the model was. For specific details see the Supplementary file 1, metabolomics analysis section.

Orthogonal partial least squares discriminant analysis (OPLS-DA) was modified based on partial least squares discriminant analysis (PLS-DA) to filter out noise irrelevant to classification information, and to improve the analytical ability and validity of the model. Similar to the PLS-DA model in the OPLS-DA model, the model evaluation parameters (R2Y, Q2) obtained through 7-fold cross-validation, and the closer R2Y and Q2 were to 1, the more stable and reliable the model was. In OPLS-DA model permutation test, the OPLS-DA model was established by 200 permutations to obtain R2 and Q2 values of the random model by randomly changing the order of the classification variable Y, which indicated that the model is robust and reliable, and no fitting had occurred. For specific details see the Supplementary file 1, metabolomics analysis section.

### Network analysis

Protein-protein interaction networks (PPI) of differentially expressed genes in KEGG pathways were analyzed with the String software (https://stringdb.org/cgi/input.pl), Spearman correlation analysis was used to analyze the correlation coefficient (r) between differentially expressed genes and metabolites. The PPI visual analysis and correlation network of these differentially expressed genes and metabolites were performed with the defined correlation coefficient (r ≤ − 0.40 or r ≥ 0.40) using software Cytoscape (Version 3.6.1, https://cytoscape.org/) [33]. Other statistical analyses were carried out using the statistical package SPSS 21.0 (SPSS Inc., Chicago, IL. USA). Differences between groups were tested using the t-test for independent samples. Significant differences were defined as *P* < 0.05, and the data were presented as the means ± standard deviation (SD).

## Supplementary Information

**Additional file 1: Table S1.** design and synthesis of the gene primers by Invitrogen Biotechnology Co., Ltd. (Shanghai, China); Table S2: raw sequence characteristics and mapping to the jungle fowl genome Gallus_gallus-5.0.; Table S3: compositions of diets and housing details; Normalization of the gene count matrix; Metabolomics analysis.

**Additional file 2.** Up- and down regulated genes, and genes associated with melanogenesis.

**Additional file 3.** Metabolite abundances: Table S4–6: positive ion mode; Table S7–9: negative ion mode.

**Additional file 4.** Correlation coefficient between DEGs and SDM (or DMs) – Tables Positive and Negative ion mode.

**Additional file 5.** Full length western blot.

## Data Availability

The RNA-Seq raw data have been deposited in the Genome Sequence Archive of the National Genomics Data Center, China National Center for Bioinformation/Beijing Institute of Genomics, Chinese Academy of Sciences, under accession number CRA004775 (https://ngdc.cncb.ac.cn/gsa/s/W0cL8Y79).

## Abbreviations

DEGs: Differentially expressed genes
PCA: Principal component analysis
KEGG: Kyoto Encyclopedia of Genes and Genomes
OPLS DA: Orthogonal Partial Least Squares Discrimination Analysis
VIP: Variable importance in the projection
SDM: Significantly differential metabolites
DM: Differential metabolites
16:0 LYSO PE: 1-Palmitoyl-2-hydroxy-sn-glycero-3-phosphoethanolamine
13(S)-HODE: Hydroxyoctadecadienoic acid
β-NMN: beta-Nicotinamide D-ribonucleotide
MTA: S-Methyl-5′-thioadenosine
DOPC: 1,2-dioleoyl-sn-glycero-3-phosphatidylcholine
LPA: lysoph-osphatidylcholine
IMP: Inosine monophosphate
LPL: Lysophospholipid
GPCRs: G-protein coupled receptors
PLC-β: Phospholipase C-β
SSTRs: Somatostatin receptors
LC-MS/MS: Liquid chromatography-tandem mass spectrometry
