# Supplementary material for: Integrative analysis of transcriptomics and metabolomics to reveal the melanogenesis pathway of muscle and related meat characters in Wuliangshan black-boned chickens
Source: BMC Genomics. 2022 Mar 2;23:173. doi: 10.1186/s12864-022-08388-w (PMC8892760; doi:10.1186/s12864-022-08388-w)
Supplement: Supplementary file 1 — Additional file 1: Table S1. design and synthesis of the gene primers by Invitrogen Biotechnology Co., Ltd. (Shanghai, China); Table S2: raw sequence characteristics and mapping to the jungle fowl genome Gallus_gallus-5.0.; Table S3: compositions of diets and housing details; Normalization of the gene count matrix; Metabolomics analysis. [file 12864_2022_8388_MOESM1_ESM.docx]

Table S1 Primer information of DEGs

| Genes | Gene accession number | Sequence of Primers (5’->3’) | Production length (bp) | Annealing Temperature (˚C) |
| --- | --- | --- | --- | --- |
| β-actin | L08165.1 | F: GTGTGATGGTTGGTATGGGC | 58 | 225 |
|  |  | R: CTCTGTTGGCTTTGGGGTTC |  |  |
| PDZK1 | XM_416669.6 | F: CCTGCAAAGGCTGCTTCACA | 59 | 193 |
|  |  | R: TGTCTCCATCCTTCAAGCCAG |  |  |
|  |  | R: GACATGTTGAAGGAGCCGGA |  |  |
| TFAP2B | NM_204895.1 | F: ATCTATGAGGACCGGCATGA | 58 | 181 |
|  |  | R: GGTCCTGGCTTTGGTGGTAA |  |  |
| TFAP2A | NM_205094.1 | F: TCAAGTACGAGGAGTGCGAG | 59 | 198 |
|  |  | R: GGGAGTAGGGGTCCTGAGAC |  |  |
|  |  | R: GAGGCCGGAACGACTCATAG |  |  |
| MITF | NM_205029 | F: TTTGCTCCCCTGATATGGTC | 60 | 199 |
|  |  | R: CCCATTTTTGCAGGAACACT |  |  |

Table S2. Sequencing data statistics

| Sample | Raw data | | Clean data | | Clean data Ratio(%) | | Mapped Ratio(%) |
| --- | --- | --- | --- | --- | --- | --- | --- |
|  | Sequences | Bases(bp) | Sequences | Bases(bp) | Sequences | Bases |  |
| CB1 | 102194018 | 15180818120 | 101788762 | 14926988949 | 99.60 | 98.33 | 78.20 |
| CB2 | 88315022 | 13109664236 | 87980470 | 12890056081 | 99.62 | 98.32 | 75.26 |
| CB3 | 96747288 | 14260647352 | 96424166 | 14045951820 | 99.67 | 98.49 | 75.87 |
| CB4 | 99853030 | 14815112908 | 99456974 | 14565096566 | 99.60 | 98.31 | 77.11 |
| CB5 | 100835344 | 14910265942 | 100720876 | 14815081521 | 99.89 | 99.36 | 78.24 |
| CB6 | 100687630 | 14897999484 | 100521788 | 14768705358 | 99.84 | 99.13 | 74.63 |
| CH1 | 97230718 | 14555042964 | 95176732 | 14492754994 | 97.89 | 99.57 | 76.19 |
| CH2 | 97802812 | 14632196524 | 95747656 | 14569768481 | 97.90 | 99.57 | 76.19 |
| CH3 | 84452768 | 12631301126 | 82828586 | 12584688554 | 98.08 | 99.63 | 75.04 |
| CH4 | 58218954 | 8732843100 | 57383266 | 8677159321 | 98.56 | 99.36 | 89.98 |
| CH5 | 94390790 | 14122530710 | 92476312 | 14066837110 | 97.97 | 99.61 | 73.66 |
| CH6 | 91319058 | 13654637556 | 89706706 | 13608724210 | 98.23 | 98.50 | 76.44 |
| WL1 | 97856240 | 14525908012 | 97684250 | 14410626016 | 99.82 | 99.21 | 74.21 |
| WL2 | 95151322 | 14107366882 | 94965242 | 13975225529 | 99.80 | 99.06 | 73.40 |
| WL3 | 96142946 | 14253678986 | 95779562 | 14027595665 | 99.62 | 98.41 | 73.58 |
| WL4 | 100848914 | 14921316924 | 100455328 | 14672765081 | 99.61 | 98.33 | 73.27 |
| WL5 | 99128668 | 14753507410 | 98684114 | 14497914730 | 99.55 | 98.27 | 74.27 |
| WL6 | 99996776 | 14876479492 | 99546746 | 14609125199 | 99.55 | 98.20 | 74.26 |
| Total | 1701172298  (1701.17 million) | 252941317728  (235.57 Gb) | 1687327536  (1687.33 million) | 250205065185  (233.02 Gb) |  |  |  |

Table S3. Composition of the Period I and Period II (g/kg, air dry) diets used in the experiment

| Dietary Component | Period I^1^ | Period II^1^ |
| --- | --- | --- |
| Maize | 545.0 | 580.0 |
| Soy protein | 190.0 | 167.0 |
| Toasted soybean | 140.0 | 80.0 |
| Fish meal | 35.0 | 20.0 |
| Wheat bran | 30.0 | 100.0 |
| Soya oil | 25.0 | 18.0 |
| CaHPO_4_·2H_2_O | 12.4 | 12.4 |
| Stone meal | 11.7 | 11.5 |
| Lysine | 2.2 | 2.2 |
| Methionine | 1.6 | 1.6 |
| Salt | 2.1 | 2.3 |
| Minerals and vitamins mix^2^ | 5.0 | 5.0 |
|  |  |  |
| Metabolism Energy（MJ/Kg） | 12.8 | 12.6 |
| Crude protein (CP) | 205.5 | 183.5 |
| Crude fat | 54.1 | 56.5 |
| Calcium | 10.5 | 9.8 |
| Available phosphorus | 6.8 | 6.5 |
| Lysine | 13.5 | 12.1 |
| Methionine + Cysteine | 8.8 | 7.8 |

1: Period I is age 1-30 days; Period II is older than 30 days of age; 2: Supplied per kilogram of diet: antioxidant, 100 mg; biotin, 0.3 mg; vitamin A, 12,000 IU; vitamin D3, 3000 IU; vitamin E, 18.75 mg; vitamin K3, 2.65 mg; vitamin C, 12.6 mg; cyanocobalamin, 0.025 mg; folic acid, 2.2 mg; niacin, 35 mg; pyridoxine, 6 mg; riboflavin, 9 mg; thiamine, 3.0 mg; choline chloride, 600 mg; Co, 0.3 mg; Cu, 12 mg; Fe, 50 mg; I, 1 mg; Mn, 125 mg; Mo, 0.5 mg; Se, 200 μg; Zn, 60 mg.

**Normalization of the gene count matrix**

The code is (for CH vs CB):

Specific values: Sample: factor(c(rep("CH1",1), rep("CH2",1), rep("CH3",1), rep("CH4",1), rep("CH5",1), rep("CH6",1), rep("CB1",1), rep("CB2",1), rep("CB3",1), rep("CB4",1), rep("CB5",1), rep("CB6",1))); Condition: factor(c(rep("CH", 6), rep("CB", 6))); Sample Table: data.frame(Sample = as.factor(Sample), Condition = as.factor(Condition)); Row names: (sampleTable) = colnames(raw.deseq.data); deseq: DESeqDataSetFromMatrix(countData = raw.deseq.data, colData = sampleTable, design = ~Condition); deseq: deseq[ rowSums (counts(deseq)) > 1, ]; d.deseq: DESeq(deseq); dds.sizefactor: estimateSizeFactors(d.deseq);

sizeFactors(dds.sizefactor)

**Metabolomics analysis**

**PLS-DA model**

**Table 1. Evaluation parameters of PLS-DA model for positive ion mode**

| **Group** | **R2X(cum)** | **R2Y(cum)** | **Q2(cum)** |
| --- | --- | --- | --- |
| **WL_vs_CB** | **0.313** | **0.961** | **0.126** |
| **WL_vs_CH** | **0.481** | **0.991** | **0.815** |
| **CH_vs_CB** | **0.393** | **0.95** | **0.663** |

**Note: R2X represents the interpretation rate of X variable by the model, R2Y represents the explanatory rate of the model for Y variable, and Q2 represents the predictive power of the model; cum = cumulative.**

**Table 2. Evaluation parameters of PLS-DA model for negative ion mode**

| **Group** | **R2X(cum)** | **R2Y(cum)** | **Q2(cum)** |
| --- | --- | --- | --- |
| **WL_vs_CB** | **0.385** | **0.932** | **0.547** |
| **WL_vs_CH** | **0.548** | **0.999** | **0.743** |
| **CH_vs_CB** | **0.337** | **0.972** | **0.276** |

**
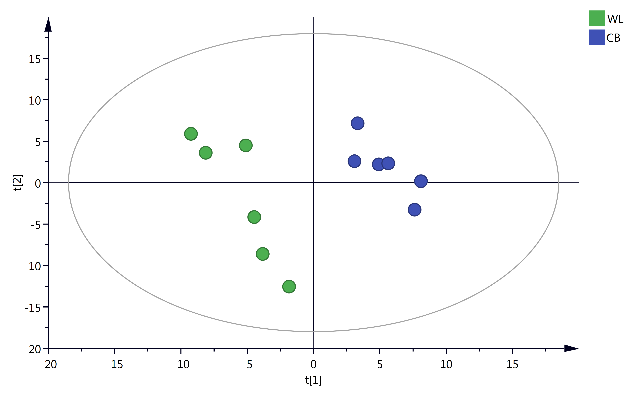

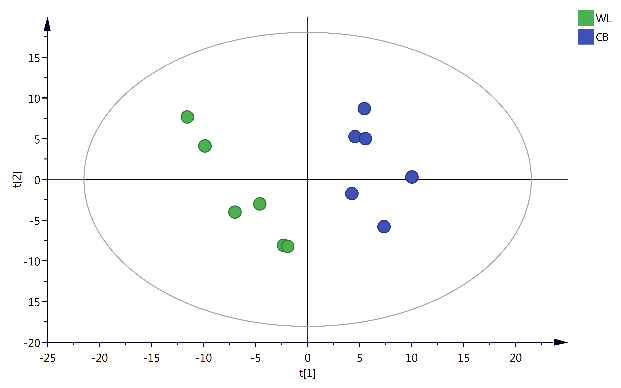
**

**A1. PLS-DA score plots of positive ion mode** **in WL vs CB A2. PLS-DA score plots of NEG mode in WL vs CB**

**
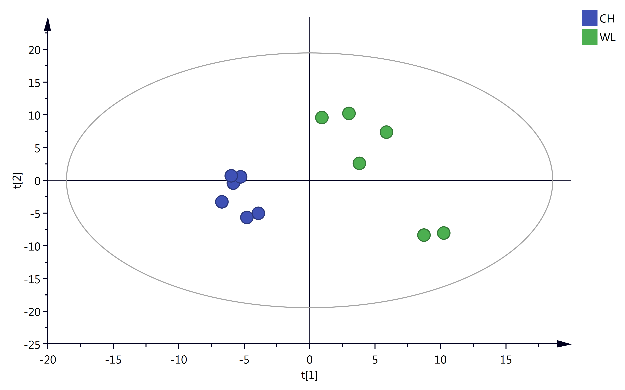

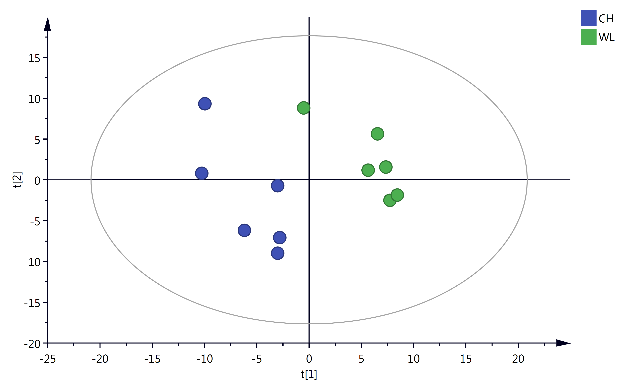
**

**B1. PLS-DA score plots of POS mode in WL vs CH B2. PLS-DA score plots of NEG mode in WL vs CH**

**
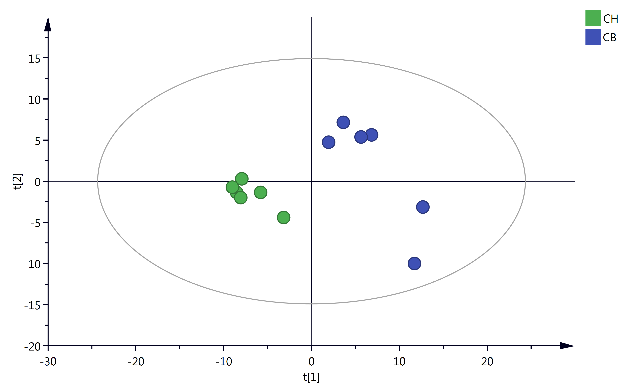

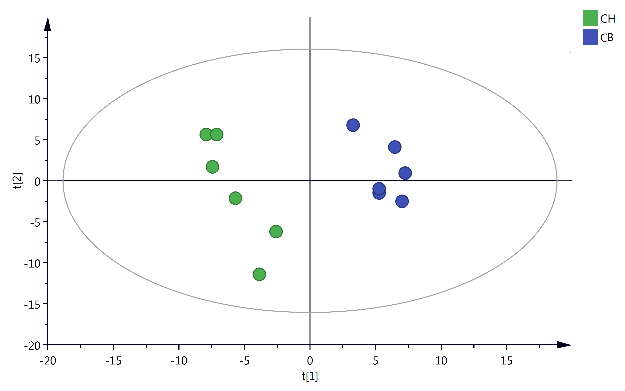
**

**C1. PLS-DA score plots of POS mode in CH vs CB C2. PLS-DA score plots of NEG mode in CH vs CB**

**Figure 1. PLS-DA score plots of positive ion** **mode (POS mode) and negative ion mode (NEG mode) in different groups and comparisons.**

**OPLS-DA model**

**Table 3. Evaluation parameters of OPLS-DA model for positive ion mode**

| **Group** | **R2X(cum)** | **R2Y(cum)** | **Q2(cum)** | **R2_intercept_** | **Q2_intercept_** |
| --- | --- | --- | --- | --- | --- |
| **WL_vs_CB** | **0.313** | **0.961** | **0.329** | **0.934** | **-0.114** |
| **WL_vs_CH** | **0.799** | **1** | **0.728** | **1** | **0.279** |
| **CH_vs_CB** | **0.393** | **0.95** | **0.673** | **0.906** | **-0.221** |

**Note: R2X represents the interpretation rate of the X variable by the model, R2Y represents the explanatory rate of the model for the Y variable, and Q2 represents the predictive power of the model. R2_Intercept_ and Q2_Intercept_ represents the intercepts of R2 and Q2 regression lines to the Y-axis.**

**
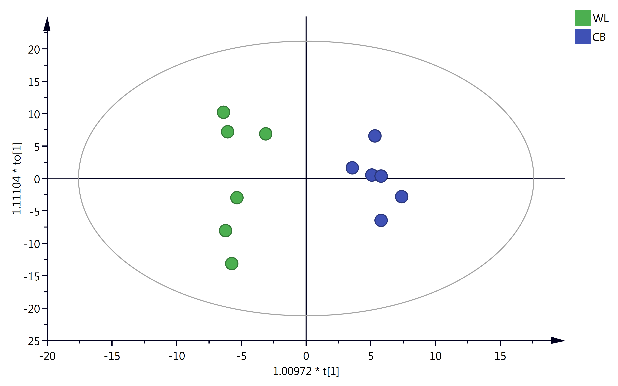

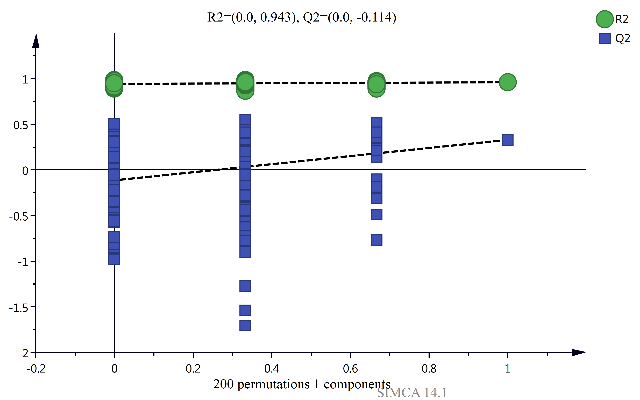
** **A1. OPLS-DA score plots of POS mode in WL vs CB A2. OPLS-DA permutation test for POS mode in WL vs CB**

**
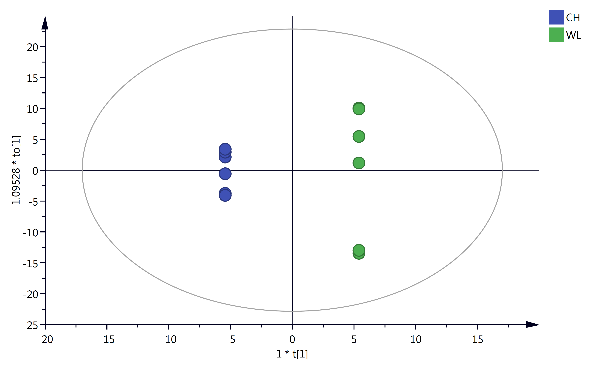

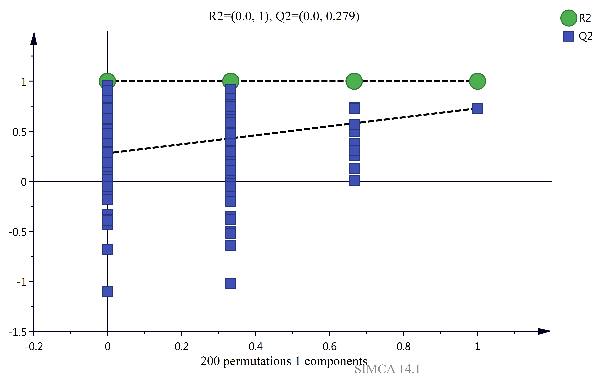
**

**B1. OPLS-DA score plots of POS mode in WL vs CH B2. OPLS-DA permutation test for POS mode in WL vs CH**

**
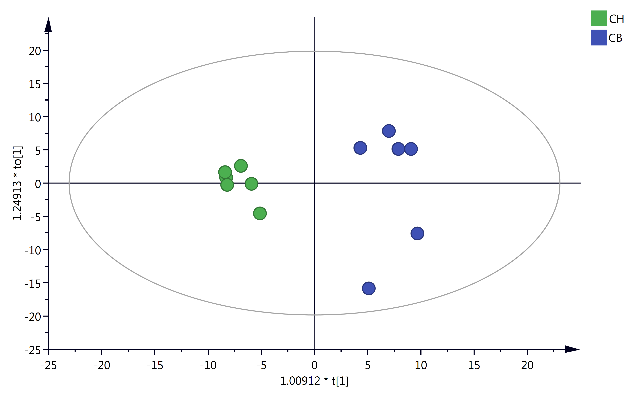

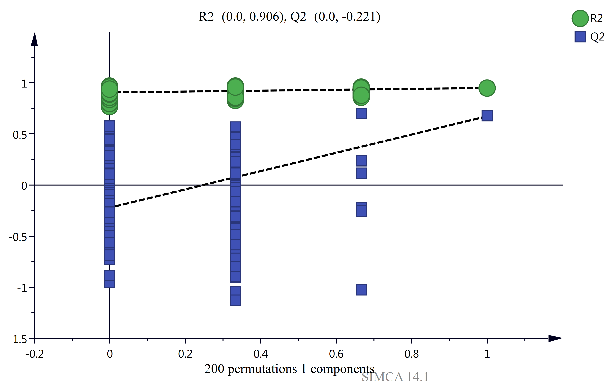
**

**C1. OPLS-DA score plots of POS mode in CH vs CB C2. OPLS-DA permutation test for POS mode in CH vs CB**

**Figure 2. OPLS-DA score plots and permutation tests for the positive ion mode (POS mode) in different groups and comparisons**

**Table 4. Evaluation parameters of OPLS-DA model for negative ion mode**

| **Group** | **R2X(cum)** | **R2Y(cum)** | **Q2(cum)** | **R2_intercept_** | **Q2_intercept_** |
| --- | --- | --- | --- | --- | --- |
| **WL_vs_CB** | **0.773** | **1** | **0.655** | **1** | **0.255** |
| **WL_vs_CH** | **0.366** | **0.924** | **0.422** | **0.935** | **-0.121** |
| **CH_vs_CB** | **0.337** | **0.972** | **0.584** | **0.915** | **-0.234** |

**
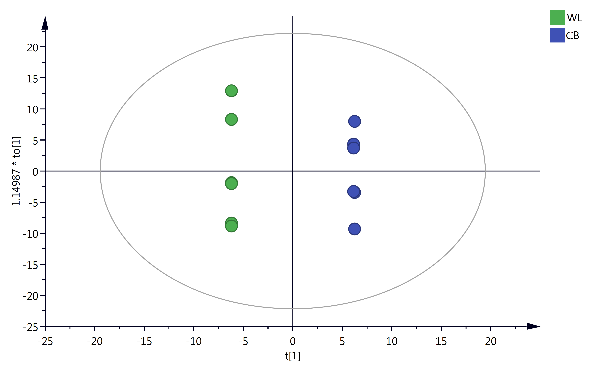

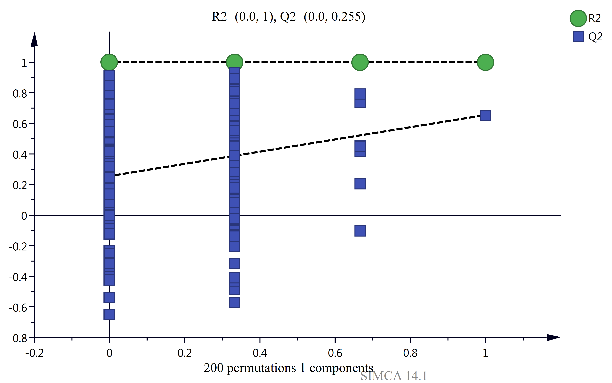
**

**A1. OPLS-DA score plots of NEG mode in WL vs CB A2. OPLS-DA permutation test for NEG mode in WL vs CB**

**
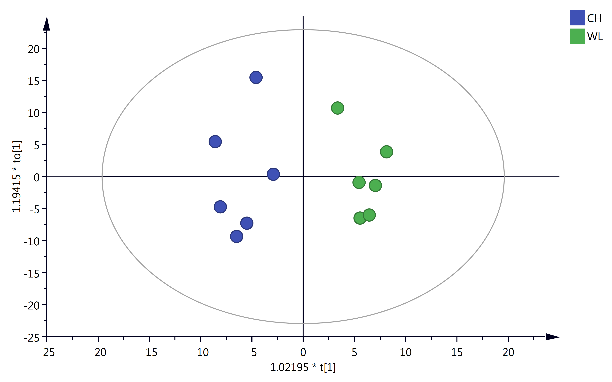

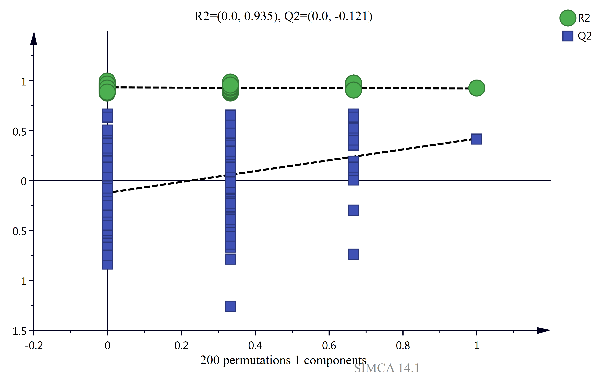
**

**B1. OPLS-DA score plots of NEG mode in WL vs CH B2. OPLS-DA permutation test for NEG mode in WL vs CH**

**
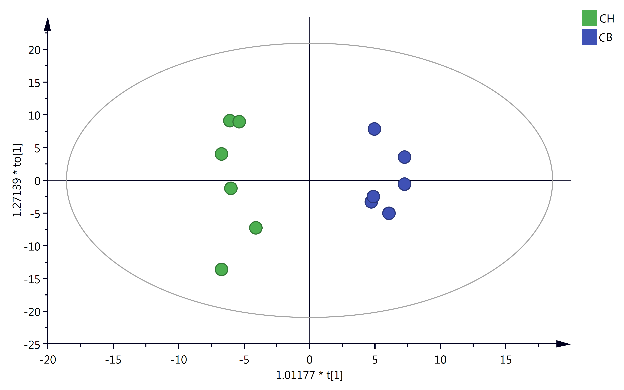

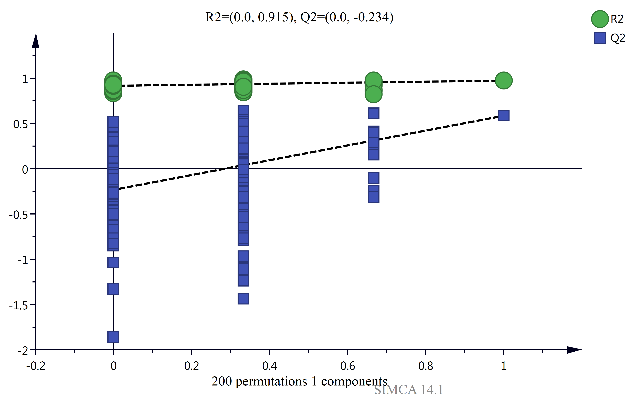
**

**C1. OPLS-DA score plots of NEG mode in CH vs CB C2. OPLS-DA permutation test for NEG mode in CH vs CB**

**Figure 3. OPLS-DA score plots and permutation tests for negative ion mode (NEG mode) in different group**
