# Supplementary material for: Integrative analysis of transcriptomics and metabolomics to reveal the melanogenesis pathway of muscle and related meat characters in Wuliangshan black-boned chickens
Source: BMC Genomics. 2022 Mar 2;23:173. doi: 10.1186/s12864-022-08388-w (PMC8892760; doi:10.1186/s12864-022-08388-w)
Supplement: Supplementary file 3 — Additional file 3. Metabolite abundances: Table S4–6: positive ion mode; Table S7–9: negative ion mode. [file 12864_2022_8388_MOESM3_ESM.docx]

Table S4. Differential abundant metabolites: WLS vs CB group in positive mode

| Metabolite | VIP | Fold change | p-value |
| --- | --- | --- | --- |
| Hypoxanthine | 9.8458 | 2.38 | 0.0476 |
| β-NMN | 1.6791 | 1.78 | 0.0018 |
| Adenine | 1.2948 | 1.75 | 0.0128 |
| 16:0 LYSO PE | 1.2671 | 1.54 | 0.0039 |
| MTA | 3.2372 | 1.45 | 0.0702 |
| L-Arginine | 3.3885 | 1.43 | 0.0699 |
| L-Tyrosine | 1.2267 | 1.28 | 0.0235 |
| Uracil | 1.7706 | 1.26 | 0.0385 |
| Nicotinamide | 9.7000 | 1.16 | 0.0655 |
| DOPC | 3.9425 | -2.23 | 0.0001 |
| Linoleic acid | 1.1065 | -2.13 | 0.0057 |
| Ile-Thr | 1.1190 | -2.06 | 0.0568 |
| Anthranilic acid | 1.7373 | -2.05 | 0.0636 |
| MG (18:2(9Z,12Z) | 1.7888 | -1.95 | 0.0120 |
| Pantothenate | 2.1608 | -1.86 | 0.0087 |
| L-Palmitoylcarnitine | 2.7352 | -1.81 | 0.0783 |
| Thioetheramide-PC | 2.2630 | -1.79 | 0.0685 |
| Pro-Phe | 1.1098 | -1.70 | 0.0209 |
| Leu-Ala | 1.0236 | -1.69 | 0.0433 |
| Phosphorylcholine | 2.0527 | -1.65 | 0.0447 |
| SOPC | 2.9608 | -1.64 | 0.0959 |
| Sphingomyelin | 2.0294 | -1.62 | 0.0747 |
| PC (16:0/16:0) | 1.6159 | -1.52 | 0.0349 |
| Sphingosine | 1.2178 | -1.49 | 0.0716 |
| L-Carnosine | 2.8067 | -1.41 | 0.0355 |
| Taurine | 2.5324 | -1.31 | 0.0038 |
| L-Carnitine | 1.2877 | -1.18 | 0.0350 |

| Table S5. Differential abundant metabolites: WLS vs CH group in positive mode | | | |
| --- | --- | --- | --- |
| Metabolites | VIP | Fold change | p-value |
| Thioetheramide-PC | 3.3345 | 2.06 | 0.0127 |
| 16:0 LYSO PE | 1.3031 | 1.85 | 0.0192 |
| Allopurinol riboside | 3.6771 | 1.79 | 0.0589 |
| β-NMN | 1.1955 | 1.65 | 0.0280 |
| Glycerophosphocholine | 8.3513 | 1.60 | 0.0220 |
| Erucamide | 2.2746 | 1.52 | 0.0919 |
| Hypoxanthine | 6.6627 | 1.42 | 0.0447 |
| Inosine | 3.7446 | 1.42 | 0.0763 |
| Taurine | 2.1017 | 1.20 | 0.0699 |

Table S6. Differential abundant metabolites: CH vs CB group in positive mode

| Metabolites | VIP | Fold change | p-value |
| --- | --- | --- | --- |
| MTA | 2.8080 | 1.38 | 0.0673 |
| Creatine | 2.0152 | 1.12 | 0.0100 |
| β-NMN | 1.0289 | 1.08 | 0.0192 |
| Inosine | 1.4016 | -1.16 | 0.0414 |
| Acetylcholine | 4.7299 | -1.16 | 0.0978 |
| L-Carnitine | 1.2720 | -1.20 | 0.0118 |
| Allopurinol riboside | 1.1767 | -1.21 | 0.0409 |
| L-Carnosine | 1.9071 | -1.35 | 0.0282 |
| Lys-Ile | 1.4237 | -1.43 | 0.0047 |
| S-lysoPC | 2.4572 | -1.45 | 0.0960 |
| PC(16:0/16:0) | 1.6814 | -1.53 | 0.0997 |
| Choline | 3.1250 | -1.56 | 0.0303 |
| Taurine | 3.2268 | -1.57 | 0.0004 |
| Erucamide | 1.9587 | -1.58 | 0.0973 |
| SOPC | 1.8013 | -1.60 | 0.0753 |
| Glycerophosphocholine | 8.5962 | -1.80 | 0.0107 |
| Sphingosine | 1.2916 | -1.84 | 0.0112 |
| MG(18:2(9Z,12Z) | 1.3327 | -1.84 | 0.0430 |
| Anthranilic acid | 1.4538 | -1.86 | 0.0748 |
| Leu-Ala | 1.0315 | -1.91 | 0.0053 |
| Hypoxanthine | 2.8286 | -1.96 | 0.0576 |
| DOPC | 3.2905 | -1.97 | 0.0001 |
| Pantothenate | 2.1482 | -2.06 | 0.0039 |
| L-Palmitoylcarnitine | 2.5780 | -2.38 | 0.0445 |
| Ile-Thr | 1.0511 | -2.48 | 0.0132 |
| Thioetheramide-PC | 2.6428 | -2.64 | 0.0341 |
| Linoleic acid | 1.0444 | -3.00 | 0.0008 |

Table S7. Differential metabolite from WLS vs CB group in negative mode

| Description | VIP | Fold change | p-value |
| --- | --- | --- | --- |
| Uracil | 3.8087 | 2.35 | 0.0659 |
| MTA | 1.6424 | 2.15 | 0.0763 |
| ketoisocaproic acid | 3.4463 | 1.96 | 0.0239 |
| 13(S)-HODE | 3.4698 | 1.84 | 0.0184 |
| 16:0 LYSO PE | 1.5047 | 1.65 | 0.0011 |
| Hypoxanthine | 8.3687 | 1.65 | 0.0178 |
| Xanthine | 1.3595 | 1.53 | 0.0681 |
| Inosine | 1.1292 | 1.38 | 0.0726 |
| N-Acetyl-L-aspartic acid | 1.3414 | 1.37 | 0.0550 |
| Adenine | 1.3685 | 1.34 | 0.0183 |
| ADP | 2.2283 | 1.27 | 0.0729 |
| Uridine | 3.9978 | 1.21 | 0.0193 |
| L-Glutamate | 1.6746 | -1.14 | 0.0683 |
| Taurine | 2.8232 | -1.25 | 0.0139 |
| Palmitic acid | 7.3213 | -1.30 | 0.0205 |
| Phosphorylcholine | 1.2322 | -1.32 | 0.0834 |
| Myristic acid | 2.4230 | -1.37 | 0.0310 |
| Linoleic acid | 10.2940 | -1.39 | 0.0025 |
| Pantothenate | 3.8793 | -1.45 | 0.0063 |
| L-Glutamine | 1.5954 | -1.45 | 0.0650 |
| Eicosapentaenoic acid | 2.1673 | -1.53 | 0.0055 |
| Glutathione | 2.5775 | -1.54 | 0.0145 |
| Myristoleic acid | 1.5148 | -1.74 | 0.0157 |
| cis-9-Palmitoleic acid | 5.8589 | -1.83 | 0.0171 |
| Oleic acid | 2.0452 | -2.45 | 0.0371 |
| C18:3 (cis-6,9,12) | 5.7163 | -2.84 | 0.0001 |

Table S8. Differential metabolite from WLS vs CH group in negative mode

| Description | VIP | Fold change | p-value |
| --- | --- | --- | --- |
| Stearic acid | 4.3591 | 2.71 | 0.0013 |
| 13(S)-HODE | 2.4992 | 1.94 | 0.0064 |
| 16:0 LYSO PE | 1.6223 | 1.87 | 0.0182 |
| Phosphorylcholine | 1.3396 | 1.55 | 0.0043 |
| DGLA, 20:3 (n=6) | 3.8204 | 1.46 | 0.0040 |
| Norethindrone Acetate | 2.9289 | 1.42 | 0.0344 |
| Inosine | 11.3763 | 1.41 | 0.0647 |
| DEEP (16:0 PE) | 1.2084 | 1.27 | 0.0332 |
| Uridine | 3.8744 | 1.24 | 0.0316 |
| 2-Oxoadipic acid | 2.5794 | 1.08 | 0.0318 |
| Creatine | 1.2304 | -1.10 | 0.0134 |
| Caprylic acid | 1.3799 | -1.12 | 0.0186 |
| Pelargonic acid | 1.8755 | -1.15 | 0.0026 |
| NANA | 1.0744 | -1.52 | 0.0634 |

Table S9. Differential metabolite from CH vs CB group in negative mode

| description | VIP | Fold change | p-value |
| --- | --- | --- | --- |
| MTA | 1.7372 | 1.87 | 0.0375 |
| ketoisocaproic acid | 2.7011 | 1.48 | 0.0795 |
| NANA | 1.0630 | 1.42 | 0.0807 |
| Adenine | 1.7773 | 1.38 | 0.0455 |
| D-Mannitol | 1.3151 | 1.26 | 0.0637 |
| Caprylic acid | 1.6008 | 1.17 | 0.0643 |
| Pelargonic acid | 2.2297 | 1.17 | 0.0112 |
| Creatine | 2.3689 | 1.06 | 0.0331 |
| Inosine | 4.6587 | -1.16 | 0.0637 |
| L-Carnosine | 8.7151 | -1.17 | 0.0352 |
| L-Glutamate | 2.5729 | -1.23 | 0.0055 |
| CADPR | 1.1696 | -1.25 | 0.0109 |
| Creatinine | 1.5274 | -1.30 | 0.0824 |
| DEEP (16:0 PE) | 2.2071 | -1.40 | 0.0061 |
| NAD | 1.1007 | -1.45 | 0.0014 |
| Taurine | 4.5628 | -1.48 | 0.0005 |
| Benzoic acid | 1.2647 | -1.49 | 0.0412 |
| Palmitic acid | 12.0462 | -1.50 | 0.0013 |
| L-Anserine | 3.2208 | -1.51 | 0.0339 |
| L-Carnitine | 1.0124 | -1.54 | 0.0231 |
| D-Ribose 5-phosphate | 1.5964 | -1.56 | 0.0088 |
| Pantothenate | 4.7880 | -1.59 | 0.0025 |
| Linoleic acid | 1.4391 | -1.60 | 0.0708 |
| Myristic acid | 3.8083 | -1.67 | 0.0033 |
| DGLA, 20:3 (n=6) | 5.6342 | -1.71 | 0.0018 |
| Eicosapentaenoic acid | 2.8060 | -1.73 | 0.0017 |
| Myristoleic acid | 2.0571 | -1.95 | 0.0016 |
| Glutathione | 3.8885 | -1.97 | 0.0010 |
| Phosphorylcholine | 2.3140 | -2.05 | 0.0016 |
| Stearic acid | 3.3998 | -2.05 | 0.0729 |
| C18:3 (cis-6,9,12) | 6.2737 | -2.49 | 0.0004 |
| Oleic acid | 2.2195 | -2.82 | 0.0387 |
| cis-9-Palmitoleic acid | 9.4643 | -2.89 | 0.0003 |

**Abbreviations**

16:0 LYSO PE: 1-Palmitoyl-2-hydroxy-sn-glycero-3-phosphoethanolamine, β-NMN: beta-Nicotinamide D-ribonucleotide, MTA: S-Methyl-5'-thioadenosine, 13(S)-HODE: Hydroxyoctadecadienoic acid, DEEP (16:0 PE): sn-Glycerol 3-phosphoethanolamine, SOPC: 1-Stearoyl-2-oleoyl-sn-glycerol 3-phosphocholine (SOPC), C18:3 (cis-6,9,12): all cis-(6,9,12)-Linolenic acid, ADP: Adenosine 5'-diphosphate (ADP), NAD: Nicotinamide adenine dinucleotide (NAD), DGLA, 20:3 (n=6): Dihomo-gamma-Linolenic Acid, 20:3 (n=6), NANA: N-Acetylneuraminic acid, DOPC: 1,2-dioleoyl-sn-glycero-3-phosphatidylcholine (18:1 PC (CIS), S-lysoPC: 1-Stearoyl-2-hydroxy-sn-glycero-3-phosphocholine, CADPR: Cyclic adenosine diphosphate ribose, GPC: Glycerophosphocholine.
