# Supplementary material for: Integrative analysis of transcriptomics and metabolomics to reveal the melanogenesis pathway of muscle and related meat characters in Wuliangshan black-boned chickens
Source: BMC Genomics. 2022 Mar 2;23:173. doi: 10.1186/s12864-022-08388-w (PMC8892760; doi:10.1186/s12864-022-08388-w)
Supplement: Supplementary file 5 — Additional file 5. Full length western blot. [file 12864_2022_8388_MOESM5_ESM.docx]

Supplementary: Full-length gels and blots.


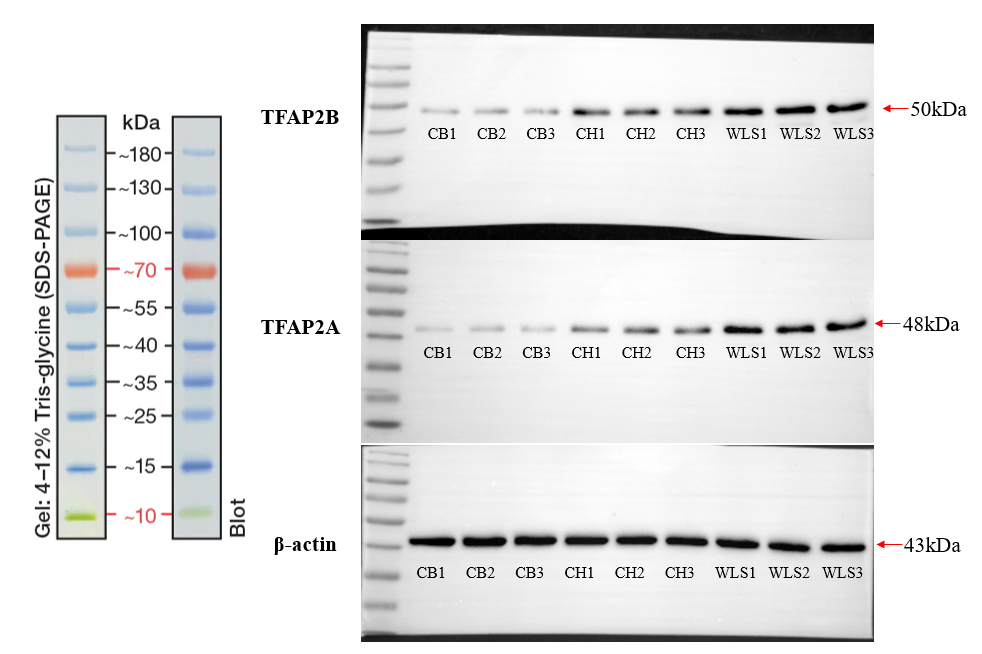


The electrophoresis time is relatively short, so the whole picture appears to be small. The original image has included the full maker range from 15kDa to 180kDa, the minimum 10kDa portion of the lower edge has been cut down after electrophoresis

, and the upper edge is fully included. Before hybridization with antibody, the original spot was not cut, only the upper concentrated glue part was removed, and all the separated glue part was retained.


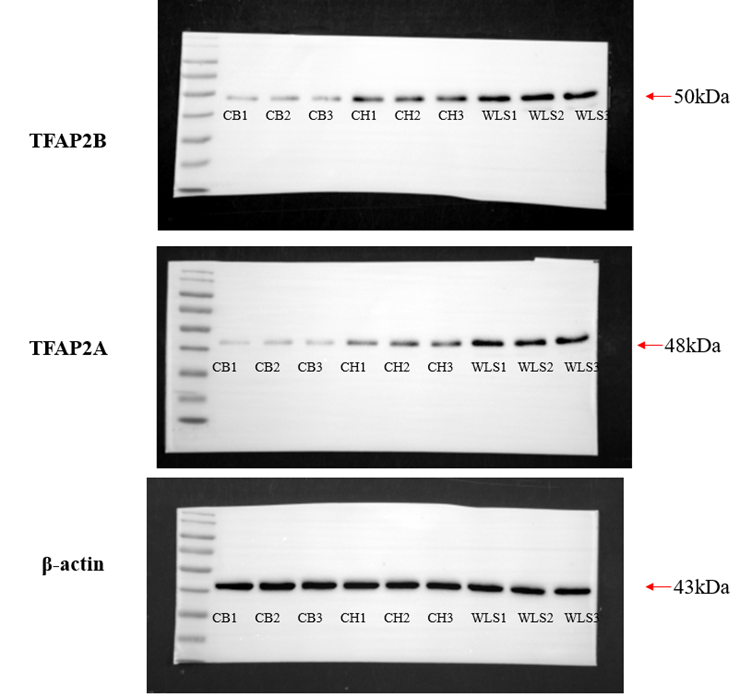


**For Figure 6C：**

We did western-blot twice. The results for the first time:


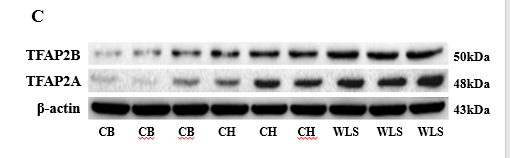


The results for the second time:


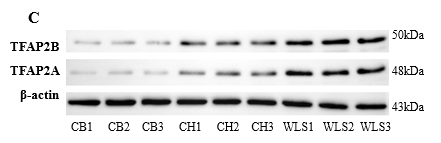


For Figure 6B, we used the second time experiment.
